# An Examination of Distractor Susceptibility of Prioritized and Unprioritized Information in Visual Working Memory

**DOI:** 10.5334/joc.462

**Published:** 2025-09-25

**Authors:** Evie Vergauwe, Caro Hautekiet, Naomi Langerock

**Affiliations:** 1 Faculty of Psychology and Educational Sciences, University of Geneva, Geneva, Switzerland; 2 Vrije Universiteit Amsterdam, The Netherlands

**Keywords:** WM, visual WM, attention, focus of attention, distraction

## Abstract

This report presents three behavioral experiments examining how different approaches of attentional prioritization influence distractor susceptibility in visual working memory (WM). We used three prioritization approaches: spontaneous, cued-based, and reward-based. In Experiments 1a and 1b, which involved spontaneous prioritization, we found that the distractor susceptibility of the last memory item – often assumed to be in the focus of attention – did not differ from that of other items in WM. In Experiment 2, cue-based prioritization was associated with reduced distractor susceptibility for the cued item, whereas reward-based prioritization showed no such effect for the highly-rewarded item, regardless of when the priority signal was presented (before, during, or after encoding). Thus, across these three experiments, prioritization was found to either reduce or have no effect on distractor susceptibility, but never to increase it. This dataset provides a basis for further investigation into the interaction between attention and interference in WM under different prioritization approaches.

Selective attentional focusing on the most relevant mental representations has been proposed as a key mechanism for prioritizing information within working memory (WM; e.g., Allen, 2019; Gazzaley & Nobre, 2012; Myers et al., 2017; Oberauer, 2019; Souza & Oberauer, 2016). The present study investigates how focused attention affects the distractor susceptibility of prioritized representations in WM.

Prior research on prioritization in WM often uses the retro-cue paradigm (Griffin & Nobre, 2003; Landman et al., 2003), where a cue indicates the memory item most likely to be tested. Retro-cues (i.e., cues shown after encoding) typically improve memory performance for the cued item over uncued items (see Souza & Oberauer, 2016, for a review). The cue is thought to direct the focus of attention to the representation of the item presented at the cued location, thereby prioritizing that representation within WM. Many studies show that the cued item is also less susceptible to distractors (e.g., Barth & Schneider, 2018; Makovski & Jiang, 2007; Schneider et al., 2017; Souza et al., 2016; van Moorselaar et al., 2015; Zhang & Lewis-Peacock, 2023a), suggesting protection within the focus of attention (Jonides et al., 2008; Oberauer & Lin, 2017; Pertzov et al., 2013; Souza & Oberauer, 2016).

However, other findings challenge this view. In particular, Hu et al. (2014) showed that the last-presented item, assumed to reside in the focus of attention, was more disrupted by perceptual interference than other list items. This suggests that focused attention may increase, rather than reduce, distractor susceptibility (see also Allen & Ueno, 2018; Hitch et al., 2018; Hu et al., 2016). Because of the theoretical importance of this finding, we decided to follow up on Hu et al. (2014)’s findings, as a first step to better understand how focused attention modulates distractor susceptibility. In Experiment 1, we closely followed Hu et al. (2014) to examine the distractor susceptibility of the last-presented item versus other list items. Additionally, we varied the delay after the last item to track changes in its distractor susceptibility over time.

## Experiment 1

### Method

#### Participants and Design

Participants were University of Geneva undergraduates with normal or corrected-to-normal vision, tested individually in the lab for partial course credit. In Experiment 1a, Distraction (Suffix vs. No suffix) and Delay (Short, Medium, or Long) were manipulated within subjects, with 30 participants (22 women, 8 men; mean age = 21.04 years). In Experiment 1b, Distraction was again manipulated within subjects, while Delay was manipulated between subjects, with 99 participants (78 women, 21 men; mean age = 21.22 years)^1^ divided into Short (33 participants), Medium (32 participants), and Long (34 participants) delay groups. Sample size reflect our aim to test about 30 participants per experiment or between-subjects condition. All participants provided informed consent, and the study was approved by a University of Geneva ethical committee.

#### Materials and Procedure

**Figure 1:**
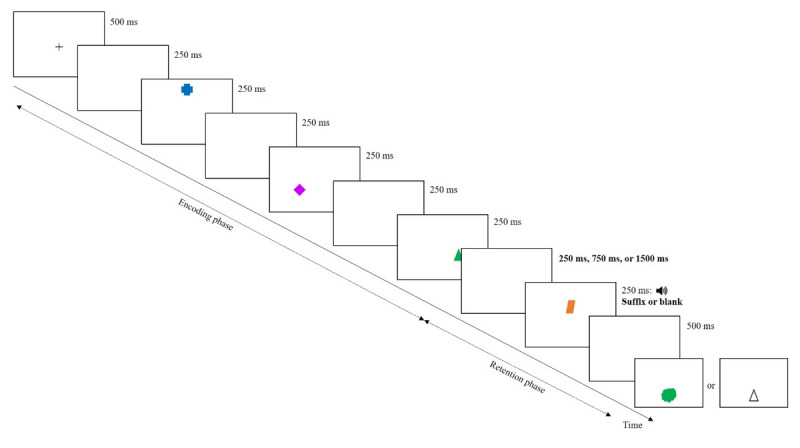
Schematic illustration of a trial in Experiment 1.

The task (Figure 1) was administered using E-prime software (Psychology Software Tools). Participants memorized series of three colored shapes (approximately 2 × 2 cm), displayed on a white background and viewed from ~50 cm. Unlike Hu et al. (2014), who presented four memory items, we used three because of low memory performance with four items in pilot tests.^2^

On each trial, three memory items were drawn from a set of 64 unique color-shape combinations (8 colors × 8 shapes; see Supplementary materials). Each of the 64 colored shapes had an equal chance of being selected, but none of the colors and shapes was repeated within a trial. On trials with distraction, a colored shape from the same set was presented (i.e., plausible suffix), with neither its color nor shape matching any of the memory items. At the end of the trial, memory was tested as in Hu et al.’s (2014) Experiment 1, using either a color patch or a colorless shape as the probe, appearing just below the center of the screen. Participants were required to recall the associated feature orally.

Each trial began with a central 500-ms fixation cross, followed by a 250-ms blank screen. Next, three colored shapes were presented sequentially, at the corners of an invisible triangle. The center of this invisible triangle was 3.5 cm above the center of the screen and the center-to-center distance between items was approximately 7 cm. Each item appeared in a random spatiotemporal sequence (i.e., serial position and spatial location were independently determined), and was displayed for 250 ms. The first two memory items were each followed by a 250-ms blank screen. The events that followed the offset of the third memory item depended on the experimental condition: (1) a 250-ms blank screen, followed by a 250-ms suffix, followed by a blank screen for 500 ms (i.e., Short delay-Suffix condition; as in Hu et al., 2014), (2) a 750-ms blank screen, followed by a 250-ms suffix, followed by a blank screen for 500 ms (i.e., Medium delay-Suffix condition), (3) a 1500-ms blank screen, followed by a 250-ms suffix, followed by a blank screen for 500 ms (i.e., Long delay-Suffix condition), (4) a 1000-ms blank screen (i.e., Short delay-No suffix condition; same as in Hu et al., 2014), (5) a 1500-ms blank screen (i.e., Medium delay-No suffix condition), or (6) a 2250-ms blank screen (i.e., Long delay-No suffix condition). The suffix appeared at the center of the invisible triangle. Like in Hu et al. (2014), a 250-ms auditory beep was played at the onset of the suffix, to help participants discriminate the suffix from the memory items. Finally, a test probe appeared about 2 cm below the center of the screen. Participants orally recalled the associated shape for a color patch, or the color for a colorless shape, and were encouraged to guess if unsure. In both experiments and in all conditions, the spatial position for each serial position was chosen randomly, so participants could not anticipate which of the three spatial positions they would be tested about.

Experiment 1a consisted of three blocks of 60 experimental trials; Experiment 1b of one block of 60 trials (see Supplementary materials). In both experiments, participants repeated “1–2–3” aloud (in French, about 2 digits/second),^3^ from fixation until probe presentation (see Hu et al. (2014). Practice trial details are in Supplementary materials.

### Results and Discussion

We used Bayesian statistics to assess the strength of evidence in our data, quantified by the Bayes Factor (BF) which can indicate support for the alternative (BF_10_) or null (BF_01_) hypothesis. We analyzed mean recall performance via Bayesian analyses of variance (BANOVA; Rouder et al., 2012), using the BayesFactor package in R (R Core Team, 2021; version 0.9.12–4.4) with default settings (e.g., rscaleFixed set to “medium” for the priors of the fixed effects, and 10000 iterations; see Rouder et al., 2012). For Experiment 1a, the BANOVA had Distraction (Suffix vs. No suffix), Serial Position (Memory item 1, 2, or 3), and Delay (Short, Medium, or Long) as within-subjects variables. For Experiment 1b, Delay was a between-subjects variable.

**Figure 2:**
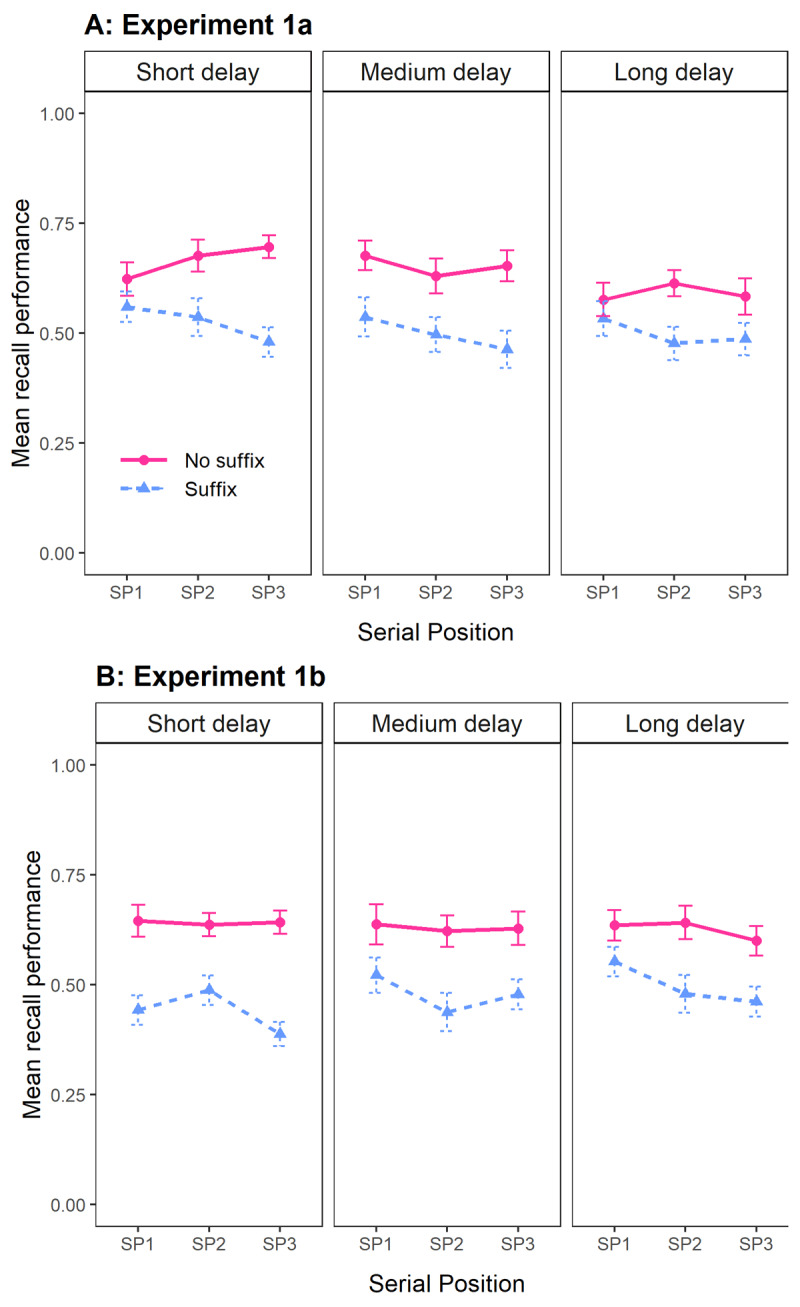
Mean recall performance in Experiments 1a **(Panel A)** and 1b **(Panel B)**, as a function of Distraction (No suffix vs. Suffix), Delay (short, medium, or long), and Serial Position (SP; memory item 1, 2, or 3). Error bars represent standard error of the mean.

In Experiment 1a, the best model included only the main effects of Distraction and Delay. As shown in Figure 2A, memory performance decreased with a suffix during retention (BF_10_ = 6.83 × 10^16^ for a main effect of Distraction). While performance appeared slightly poorer with longer delays, evidence for a main effect of Delay was inconclusive (BF_10_ = 1.70). Unlike Hu et al. (2014), who observed a recency effect, our data favored excluding Serial Position (BF_01_ = 17.81 against its main effect). Adding both Serial Position and its interaction with Distraction resulted in a model that was 20.97 times worse than the best model. The best model was also over 40’000 times better than the full model. Separate BANOVAs per Delay condition confirmed this pattern (see Supplementary materials).

In Experiment 1b, the best model only included the main effect of Distraction. As shown in Figure 2B, memory performance suffered again when a suffix was presented (BF_10_ = 1.80 × 10^26^ for a main effect of Distraction). Again, the data favored excluding a main effect of Serial Position (BF_01_ = 3.04). The data also favored excluding a main effect of Delay (BF_01_ = 11.38). Adding both Serial Position and its interaction with Distraction resulted in a model that was 29.85 times worse than the best model, and the best model was over 80’000 times better than the full model. Separate BANOVAs per Delay condition showed the same pattern (see Supplementary materials).

Experiments 1a and 1b found no evidence for heightened distractor susceptibility of the last-presented item; Distraction affected all items equally. This suggests that although the last item is vulnerable to distraction, it is not particularly so. If the last item was in the focus of attention, our data suggest items in the focus of attention are no more or less vulnerable to distraction than items outside the focus of attention. If it was not in the focus of attention, conclusions about vulnerability in the focus of attention remain uncertain. The absence of a recency effect is consistent with this, suggesting all items may have received similar focused attention during retention. Interpreting these findings is challenging because the experiments relied on spontaneous prioritization, leaving it uncertain which item was actually in the focus of attention. To address this, Experiment 2 used an explicit priority signal to indicate which item had to be prioritized.

## Experiment 2

Experiment 2 used two common prioritization approaches: cue-based, whereby attention is guided by a predictive cue, and reward-based, whereby attention is guided by high-value rewards. Cue-based studies typically show that prioritized information is less vulnerable than other information in WM (e.g., Barth & Schneider, 2018; Makovski & Jiang, 2007; van Moorselaar et al., 2015; Zhang & Lewis-Peacock, 2023b), indicating that the focus of attention shields information from interference (i.e., Protection hypothesis). In contrast, recent reward-based studies found prioritized information to be *more* vulnerable (i.e., e.g., Hu et al., 2014, 2016; Allen & Ueno, 2018; Hitch et al., 2018), suggesting that information in the focus of attention is particularly vulnerable to interference (i.e., Vulnerability hypothesis).

Whether these contrasting findings reflect genuine differences between prioritization approaches or stem from other methodological factors, such as when priority is induced (cues often appear after encoding, rewards before) or differences in memory materials and test formats, remains unclear. To address this, Experiment 2 used cue- and reward-based prioritization within the same paradigm, and varied their timing (before, during, or after encoding). Based on available evidence, we expected cue-based prioritization to support the Protection hypothesis and reward-based prioritization the Vulnerability hypothesis, with no a priori reason to expect this pattern to vary with the timing of prioritization.

### Method

#### Participants and Design

The 64 participants (53 women, 9 men, 2 non-binary; mean age = 22.71 years) were University of Geneva undergraduates with normal or corrected-to-normal vision, tested individually in the lab for partial course credit. Prioritization Type (Cue vs. Reward) was manipulated between subjects, while Prioritization Time (Baseline, Before, During, or After) and Distraction (Suffix vs. No suffix) were manipulated within subjects. Participants were assigned randomly to either the Cue or the Reward group (both 32 participants). All participants provided informed consent, and the study was approved by a University of Geneva ethical committee.

#### Materials and Procedure

**Figure 3:**
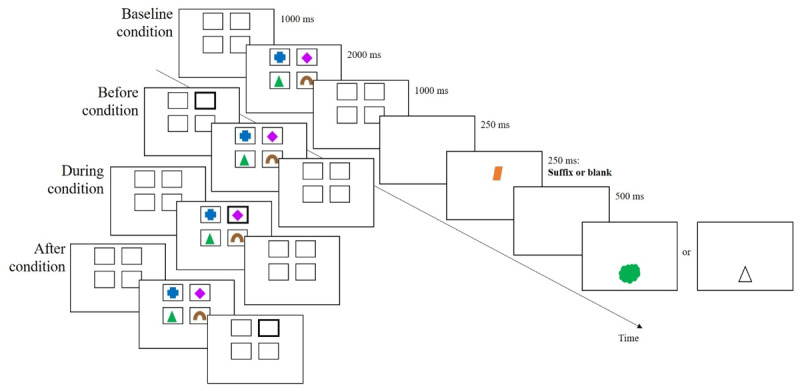
Schematic illustration of a trial in the different conditions of Experiment 2.

The task (Figure 3) was close to the task used by Allen and Ueno (2018, Experiment 1), and was administered using E-prime software. Participants memorized sets of four colored shapes (2 × 2 cm), simultaneously presented against a white background. Experiment 1’s colors and shapes were used, except black was replaced by brown to avoid overlap with the black-bordered boxes.

On each trial, four memory items were drawn from a set of 64 unique color-shape combinations. Each of the 64 colored shapes had an equal chance of being selected, but none of the colors and shapes was repeated within a trial. On trials with distraction, a colored shape from the same set was presented (plausible suffix), with neither its color nor shape matching any of the memory items. Memory was tested using the same procedure as in Experiment 1.

Each trial began with a central 500-ms fixation cross followed by three successive screens: (1) four empty boxes (3.2 × 2.6 cm), for 1000 ms, presented in two rows of two boxes (one row in the upper part of the screen and one in the lower part), (2) the same four boxes, with each box containing one memory item (2 × 2 cm), for 2000 ms, and (3) the four empty boxes again, for 1000 ms. All memory items were shown simultaneously to simplify the experimental design and data analysis (avoiding the inclusion of serial position as an additional factor).

In all conditions, boxes had thin, black borders in the three screens. In the Baseline condition, no box was highlighted. In the Before condition, one box in the first screen was highlighted by a thick, black border (i.e., before encoding). In the During condition, one box in the second screen was highlighted by a thick, black border (i.e., during encoding). In the After condition, one box in the third screen was highlighted by a thick, black border (i.e., after encoding). Trials for each of these four Prioritization Time conditions were presented in separate blocks. Within each block, the location of the highlighted box was determined randomly on each trial, with the constraint that each of the four boxes was equally often highlighted for each of the experimental conditions. On Suffix trials, a 250-ms blank screen was followed by a 250-ms suffix at the center of the screen and then a 500-ms blank screen. On No suffix trials, the 1000-ms delay remained blank. The color-tested and shape-tested trials were randomly distributed across the experiment (50% chance).

Instructions about the highlighted boxes and corresponding testing probabilities varied between the two experimental groups (see Supplementary materials). In all blocks and trials, participants in both groups repeated «1–2–3–4» aloud (in French, about 2 digits/second), from fixation until probe presentation. Practice trial details are in Supplementary materials.

### Results and Discussion

In both groups, we analyzed mean recall performance (proportion of correct responses).

**Figure 4:**
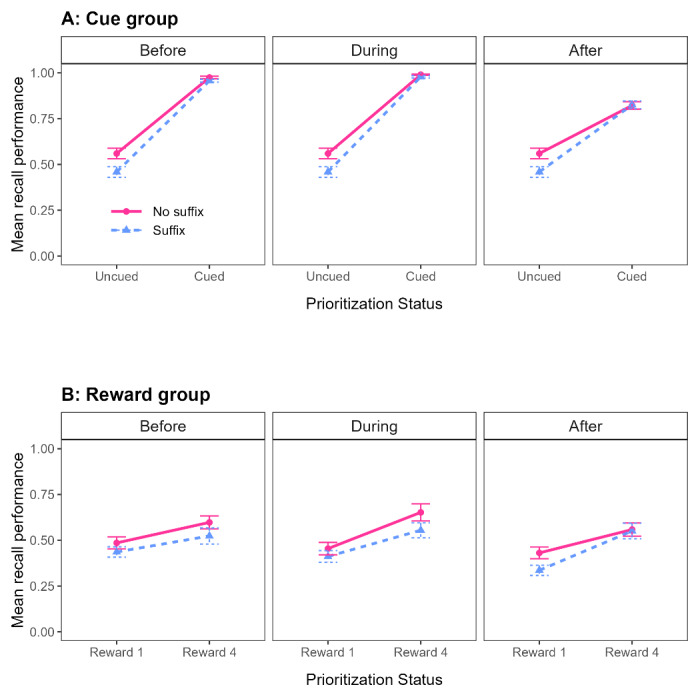
Mean recall performance in Experiment 2, as a function of Prioritization Type (Cue group in **panel A**, Reward group in **panel B**), Distraction (No suffix vs. Suffix), and Prioritization Status (Uncued vs. Cued in the Cue group, panel A; Low-reward 1 vs. Reward 4 for the conventional comparison in the Reward group, **panel B**). Error bars represent standard error of the mean. Note that in **Panel A**, unprioritized items are taken from baseline trials and thus the same values are shown in the three panels for uncued items. This allows for a direct comparison of the pattern across panels.

We analyzed the data of the Cue group using the comparison that is typically used in Cue paradigms: prioritized items correspond to cued items, from trials with a cue (Before, During, or After), whereas unprioritized items correspond to uncued items from trials without a cue (Baseline trials). We performed a BANOVA with Prioritization Time (Baseline, Before, During, or After) and Distraction (Suffix vs. No suffix) as within-subjects variables. The best model was the full model, including the main effects of Prioritization Time and Distraction, as well as their interaction. There was strong evidence for the interaction; removing it from the best model made the model 13.87 times worse. Next, we analyzed the data separately for the different Prioritization Time conditions. Each subset of data was analyzed with a BANOVA with Prioritization Status (Cued vs. Uncued) and Distraction (Suffix vs. No suffix) as within-subjects variables. In all cases, the best model was the full model, and excluding the interaction of interest made the model substantially worse (BF_10_ = 3.00, 3.65, and 28.91, for the interaction, in the Before, During, and After data-subsets, respectively). As shown in Figure 4A, cuing improved memory performance, and presenting a suffix disrupted recall. In line with our expectations, this disruptive effect was smaller for cued items than for uncued items. This pattern held regardless of when the cue was presented.

We analyzed the data of the Reward group using the approach that was most often used in the first studies using the Reward paradigm and examining distractor susceptibility (e.g., Allen & Ueno, 2018; Hitch et al., 2018; Hu et al., 2014, 2016): prioritized items are high-reward items and unprioritized items are low-reward items, both from trials with a reward pattern (Before, During, or After). This analysis is referred to as “Reward – conventional comparison”.^4^ Alternative and additional analyses can be found in Supplementary materials. We performed a BANOVA with Prioritization Time (Before, During, or After), Prioritization Status (High-reward vs. Low-reward) and Distraction (Suffix vs. No suffix) as within-subjects variables. The best model only included the main effects of Prioritization Status and Distraction, with very strong evidence for both (BF_10_ = 2.04 × 10^14^ and BF_10_ = 78.43, respectively). There was no evidence for a main effect of Prioritization Time (BF_01_ = 1.36). Including the interaction between Prioritization Status and Distraction resulted in a model that was 6.26 times worse than the best model, indicating that the suffix equally disrupted high-reward and low-reward items. Next, we analyzed the data separately for the different Prioritization Time conditions. Each subset of data was analyzed with a BANOVA with Prioritization Status (High-reward vs. Low-reward) and Distraction (Suffix vs. No suffix) as within-subjects variables. None of these showed convincing evidence for an interaction between Prioritization Status and Distraction (BF_01_ = 3.51, BF_01_ = 2.83, and BF_10_ = 1.07, for Before, During, and After trials, respectively). As shown in Figure 4B, highly rewarding an item improved its memory performance, and presenting a suffix disrupted recall. This disruptive effect was similar for high-reward and low-reward items, suggesting that rewarding an item does not drastically influence its distractor susceptibility, regardless of when the reward pattern was presented.

## Conclusion

Overall, Experiment 1 suggests that distractor susceptibility did not differ between the last item and other items, while Experiment 2 showed reduced distractor susceptibility under cue-based prioritization, but no clear effect under reward-based prioritization. In particular, our findings (1) replicate the protection of information in the focus of attention under cue-based prioritization, but (2) provide no evidence for either protection or heightened vulnerability under spontaneous or reward-based prioritization. Rather, in these cases, information inside and outside the focus of attention appeared equally susceptible to interference. Thus, we found prioritization to reduce distractor susceptibility or leave it unaffected, but never to increase it. These findings suggest that the vulnerability of items in the focus of attention may depend on how information is brought into the focus of attention. This dataset provides a basis for further investigation on how attention and interference interact in WM under different prioritization approaches. Our interpretation is detailed in Vergauwe et al. (2025).

## Data Accessibility Statement

The data and analysis scripts of the reported experiments are available on the Open Science Framework (https://osf.io/ygpjf/). A readme document on OSF provides meta-data. The experiment was not preregistered, and research materials are not shared. All decisions regarding design and analysis are transparently reported, along with all data exclusions, all manipulations and all measures that were recorded. The data were analyzed by the first author (EV), and were independently reproduced by the co-authors (NL for Experiment 1 and CH for Experiment 2). The data were collected between 2019 and 2021.

## Additional File

The additional file for this article can be found as follows:

10.5334/joc.462.s1 Supplementary File. Supplementary materials.

## Footnotes

Mean age was calculated on the basis of 96 participants, as the data of birth was missing for three participants.

To increase memory performance, we considered two options: increasing presentation time or decreasing set size. To our knowledge, there were no studies that had shown clear suffix effects in adults with presentation times slower than 500 ms per item. There was, however, a study that had shown a suffix effect in adults using a set size of three memory items (Brown et al., 2017). Therefore, we decided to use three memory items in the main experiment.

In Hu et al. (2014), participants had to remember four memory items and were required to repeat the sequence “1–2–3–4”. Because our participants had to remember only three memory items, we adapted the sequence that had to be repeated to “1–2–3”.

The same names are used as in Jeanneret, Vergauwe et al. (2025), which used a similar design with verbal memory materials, to facilitate comparisons between studies and WM domains.
