# Supplementary material for: An Examination of Distractor Susceptibility of Prioritized and Unprioritized Information in Visual Working Memory
Source: J Cogn. 2025 Sep 25;8(1):48. doi: 10.5334/joc.462 (PMC12466325; doi:10.5334/joc.462)

Supplementary materials

1. Method Experiment 1

Memory materials. A pool of eight saturated colors was selected (red, blue, yellow, green, aqua, purple, gray, and black), as well as a pool of eight shapes (circle, diamond, triangle, cross, arrow, star, flag, and arch).

Experimental trials. Experiment 1a consisted of 180 experimental trials in total. These were presented as three blocks of 60 trials, corresponding to the three Delay conditions. The order of these blocks was counterbalanced across participants. These blocks of trials were created by first constructing five mini-blocks of 12 trials, one per combination of Distraction condition (Suffix vs. No suffix), Type of probe (Color vs. Shape), and Serial position probed (serial position 1, 2, or 3). Within each mini-block, the 12 different trials were presented as follows: In Experiment 1b, the order of the 12 different trials was randomly determined. While we planned to do the same in Experiment 1a, a programming error in the Short delay condition resulted in a fixed order of the 12 trials within each mini-block within the Short delay block. In particular, in each mini-block, the first 6 trials included a suffix whereas the last 6 trials did not. Moreover, for each of these 6 trials, the first three trials probed color and the latter three shape, and for each of these three trials, the first trial probed the first serial position, the second trial probed the second serial position, and the third trial probed the third serial position. In Experiment 1a, five of these mini-blocks were presented for each delay condition (with the possibility to take a short break after each mini-block), resulting in 60 trials per Delay block. Experiment 1b consisted of one block of 60 experimental trials, i.e., 5 mini-blocks of 12 trials, all pertaining to the same Delay condition.

Practice trials. Like in Hu et al. (2014), participants were shown all shapes and color blobs with their names before the experimental trials. This was followed by a step-by-step task explanation and practice trials. The following practice trials were used. First, a set of six practice trials: 3 trials without suffix, without articulatory suppression, but with feedback, and 3 trials with suffix, without articulatory suppression, and without feedback. Next, a set of five practice trials with articulatory suppression and without feedback (i.e., trials like in the experimental session).

2. Method Experiment 2

Instructions and testing probabilities. In the Cue group, participants were told that the highlighted box indicated the spatial position of the memory item that was going to be tested at the end of the trial. This cue was 100% valid, and thus on Before, During, and After trials in the Cue group, memory was always tested for the cued item. In the Baseline trials of the Cue group, all items were equally likely to be tested (25%). In the Reward group, participants were told that the highlighted box indicated the spatial position of the memory item worth 4 points if correctly recalled (the three other items were worth only 1 point), but all memory items were equally likely to be tested (25%). In the Baseline trials of the Reward group, all items were equally likely to be tested (25%), and earned 1 point if correctly recalled. Participants were aware that these points represented simply notional rewards (see also Atkinson et al., 2022).

Experimental trials. In both groups, each participant completed 256 experimental trials, consisting of 32 trials per experimental condition resulting from crossing Prioritization Time (4 levels: Baseline, Before, During, After) and Distraction (2 levels: Suffix vs. No suffix). Trials were presented in four blocks of 64, one block per Prioritization Time condition, with the 32 Suffix trials and 32 No Suffix trials randomly intermixed within each block. Four versions of the experiment counterbalanced block order across participants according to a Latin square design.

Practice trials. Participants were shown all shapes and color blobs with their names before the experimental trials, as well as four examples of colored shapes in the same disposition as during the experimental trials. This was followed by a step-by-step task explanation and practice trials. Before each of the four experimental blocks, the conditions of that block were explained (reward or cue presented before, during, or after memory item presentation, or never) and two corresponding practice trials were performed. The following practice trials were used before the main session started. First, a set of four practice trials: 2 trials without suffix and without articulatory suppression but with feedback, and 2 trials with suffix but without articulatory suppression and without feedback. Next, a set of four practice trials with articulatory suppression, presenting a suffix on half of the trials, without feedback. Note that each experimental block in the main session was preceded by two practice trials that matched the conditions of the upcoming experimental trials.

3. Results Experiment 1

We ran additional, separate BANOVAs per Delay condition in Experiments 1a and 1b. These all confirmed the overall pattern, with the Distraction-only model being the best model at all delays. Specifically, we ran a BANOVA with Distraction (Suffix vs. No suffix) and Serial Position (Memory item 1, 2, or 3) as within-subjects variables for each subset of data defined by Delay (Short, Medium, and Long). In each of these, we found the Distraction-only model to be the best model, with strong evidence for the main effect of Distraction (BF_{10} = 9.25 x 10^7, 9.58 x 10^5, and 292.23, for the short, medium, and long delays of Experiment 1a; and 7.23 x 10^{14}, 6.07 x 10^5, and 2.49 x 10^5, for the short, medium, and long delays of Experiment 1b). There was consistent evidence against the main effect of Serial Position (BF_{01} = 13.43, 5.50, and 14.75, for the short, medium, and long delays of Experiment 1a; and 4.12, 6.27, and 1.75, for the short, medium, and long delays of Experiment 1b), and the full model including the interaction term

was consistently considerably worse than the best model (2.29, 36.01, and 43.81 times worse for the short, medium, and long delays of Experiment 1a; and 7.51, 38.86, and 7.04 times worse for the short, medium, and long delays of Experiment 1b).

4. Results Experiment 2

An alternative comparison in the Reward group. Next to the conventional comparison in the Reward group, we explored an alternative comparison for the Reward data, more similar to the Cue paradigm and to the comparison used in more recent studies using the Reward paradigm (e.g., Allen et al., 2021; Hu et al., 2023; Johnson & Allen, 2023). Prioritized items remained high-reward items from trials with a reward pattern, but unprioritized items were equal-reward items from baseline trials. This analysis is referred to as "Reward – alternative comparison". For this comparison, we performed a BANOVA with Prioritization Time (Baseline, Before, During, or After) and Distraction (Suffix vs. No suffix) as within-subjects variables, like in the Cue group. The best model included only the main effects of Prioritization Time and Distraction, and including the interaction between Prioritization Time and Distraction resulted in a model that was almost 9 times worse. Next, we again analyzed the data separately for the different Prioritization Time conditions. Each subset of data was analyzed with a BANOVA with Prioritization Status (High-reward vs. Equal-reward) and Distraction (Suffix vs. No suffix) as within-subjects variables. For each subset, the best model was the main effects-only model, and including the interaction of interest made the model somewhat worse (BF_{01} = 3.81, 3.58, and 1.1, for the interaction between Prioritization Status and Distraction, in the Before, During, and After subsets, respectively). As shown in the Figure below, the results of the alternative comparison align with those of the conventional comparison; highly rewarding an item improves its memory performance, but does not influence its distractor susceptibility. This pattern held again regardless of when the reward pattern was presented.

Figure

Mean recall performance in the Reward group of Experiment 2, as a function of Distraction (No suffix vs. Suffix), and Prioritization Status (Equal-reward 1 vs. Reward 4, using the alternative comparison). Error bars represent standard error of the mean. Note that unprioritized items are taken from baseline trials and thus the same values are shown in the three panels for Equal-reward 1 items.

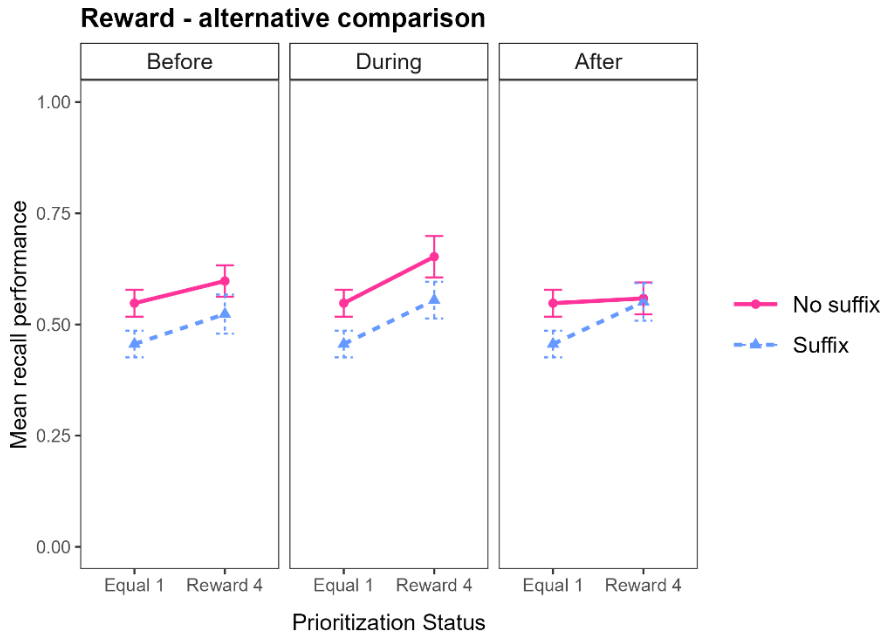

Comparison of the Cue and Reward groups. In addition, we directly compared the Cue and Reward groups by examining changes in vulnerability between prioritized and unprioritized items, using their conventional comparisons. To do so, we calculated, per participant, a vulnerability score for prioritized and unprioritized items separately (i.e., No suffix recall minus Suffix recall), using the conventional comparisons. To calculate vulnerability scores, we used the conventional comparisons described in the main text. As such, prioritized items were cued items (Cue group) or high-reward items (Reward group), both averaged across Before, During, and After trials, while unprioritized items were uncued (Cue group; Baseline trials) or low-reward items (Reward group; averaged across Before, During, and After trials). The mean vulnerability scores as shown in the figure below.

Vulnerability scores were analyzed using a BANOVA with Prioritization Status (Prioritized vs. Unprioritized) as a within-subjects variable, and Prioritization Type (Cue vs. Reward) as a between-subjects variable. The best model was the Prioritization Status-only model, which explained the data about as well as the full model including the interaction of interest (BF_{01} = 1.25, against the full model when compared to the best model).

Given the inconclusive findings regarding the interaction of interest, but apparent differences between the groups, we conducted an additional, unplanned analysis, testing the expected pattern more directly. We calculated scores that capture the difference in vulnerability scores between prioritized and unprioritized items, i.e., an alteration index of vulnerability. Per participant, vulnerability scores for unprioritized items were subtracted from vulnerability scores for prioritized items. An alteration index above 0, indicates that prioritized items are more vulnerable than unprioritized items, consistent with the Vulnerability hypothesis. An alteration index below 0 indicates that prioritized items are protected from interference, consistent with the Protection hypothesis. An index of 0 indicates equal vulnerability, implying that prioritization does not affect distractor susceptibility.

As expected, the alteration index was below 0 in the Cue group (BF_{10} = 963.43, for the corresponding one-sided t-test), supporting the Protection hypothesis. In the Reward group, the alteration index was not above 0 (BF_{01} = 5.59, for the corresponding one-sided t-test), and thus, there was evidence against the expected heightened vulnerability. In fact, the alteration index in the Reward group did not differ from 0 (BF_{01} = 5.28) and, thus prioritized and unprioritized items were equally vulnerable in the Reward group. A direct comparison of alteration indices between the Cue and Reward groups tested whether reward-based prioritization produced a larger index (above 0, indicating heightened vulnerability) than cue-based prioritization (below 0, indicating protection) and revealed modest evidence (BF_{10} = 3.48).

Figure

Mean vulnerability scores in Experiment 2 (mean recall performance in No suffix condition, minus mean recall performance in Suffix condition), as a function of Prioritization Type (Cue group on the left, Reward group on the right), and Prioritization Status (Unprioritized vs. Prioritized, corresponding to Uncued vs. Cued items, respectively, in the Cue group, and corresponding to Low-reward vs. High-reward, respectively, in the Reward group). Grey points are showing individual data, and pink lines represent the means per condition. Dotted blue line shows vulnerability score at 0. Scores above 0 indicate vulnerable representations; scores at or below 0 indicate protected representations.

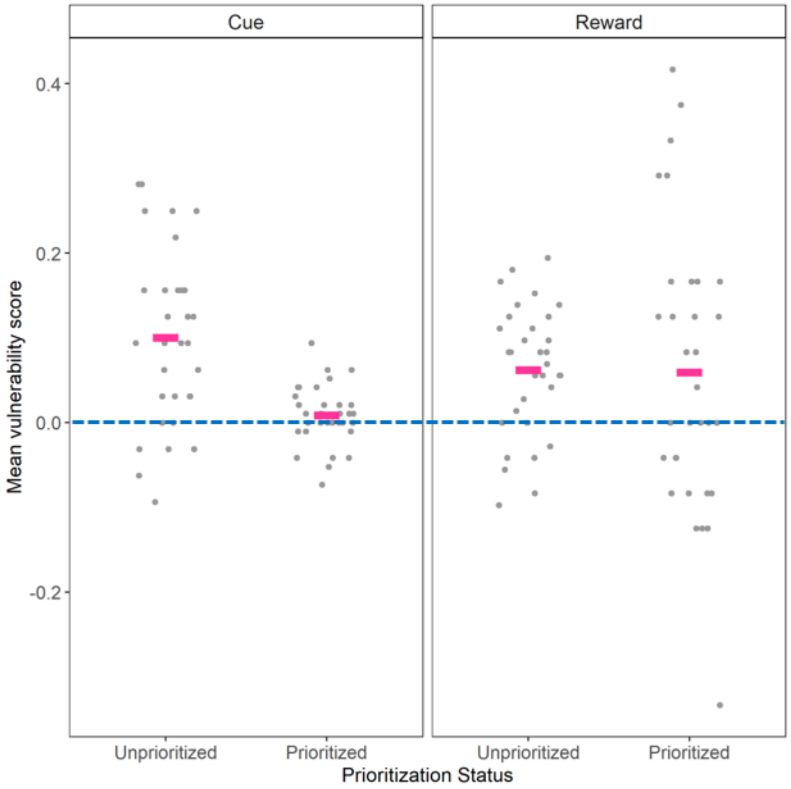

Supplement: Supplementary File. — Supplementary materials. [file joc-8-1-462-s1.pdf]
